# Correlation of a Self-Report and Direct Measure of Physical Activity Level in the Electron-Beam Tomography and Risk Assessment Among Japanese and US Men in the Post World War II Birth Cohort (ERA JUMP) Study

**DOI:** 10.2188/jea.JE20120151

**Published:** 2013-11-05

**Authors:** Marie A. Berger, Chol Shin, Kristi L. Storti, J. David Curb, Andrea M. Kriska, Vincent C. Arena, Jina Choo, Hirotsuga Ueshima, Tomonori Okamura, Katsuyuki Miura, Todd B. Seto, Kamal Masaki, Aiman El-Saed, Akira Sekikawa

**Affiliations:** 1 Department of Epidemiology, University of Pittsburgh, Graduate School of Public Health, Pittsburgh, PA, USA; 2 Department of Biostatistics, University of Pittsburgh, Graduate School of Public Health, Pittsburgh, PA, USA; 3 Institute of Human Genomic Study, Korea University, Seoul, South Korea; 4 College of Nursing, Korea University, Seoul, South Korea; 5 Department of Geriatric Medicine, University of Hawaii, John A. Burns School of Medicine, Honolulu, HI, USA; 6 Department of Medicine, University of Hawaii, John A. Burns School of Medicine, Honolulu, HI, USA; 7 Queen’s Medical Center, Honolulu, HI, USA; 8 Department of Health Sciences, Shiga University of Medical Sciences, Otsu, Japan; 9 Department of Preventive Medicine and Public Health, School of Medicine, Keio University, Tokyo, Japan

**Keywords:** ethnic groups, exercise, pedometry, self-report, occupational activity

## Abstract

**Background:**

Physical activity (PA) is complex and a difficult behavior to assess as there is no ideal assessment tool(s) that can capture all contexts of PA. Therefore, it is important to understand how different assessment tools rank individuals. We examined the extent to which self-report and direct assessment PA tools yielded the same ranking of PA levels.

**Methods:**

PA levels were measured by the Modifiable Activity Questionnaire (MAQ) and pedometer at baseline among 855 white (W), African-American (AA), Japanese-American (JA), and Korean (K) men (mean age 45.3 years) in 3 geographic locations in the ERA JUMP study.

**Results:**

Korean men were more active than W, AA, and JA men, according to both the MAQ and pedometer (MAQ total PA [mean ± SD]: 41.6 ± 17.8, 20.9 ± 9.9, 20.0 ± 9.1, and 29.4 ± 10.3 metabolic equivalent [MET] hours/week, respectively; pedometer: 9584.4 ± 449.4, 8363.8 ± 368.6, 8930.3 ± 285.6, 8335.7 ± 368.6 steps/day, respectively). Higher levels of total PA in Korean men, as shown by MAQ, were due to higher occupational PA. Spearman correlations between PA levels reported on the MAQ and pedometer indicated positive associations ranging from rho = 0.29 to 0.42 for total activity, rho = 0.13 to 0.35 for leisure activity, and rho = 0.10 to 0.26 for occupational activity.

**Conclusions:**

The 2 assessment methods correlated and were complementary rather than interchangeable. The MAQ revealed why Korean men were more active. In some subpopulations it may be necessary to assess PA domains other than leisure and to use more than 1 assessment tool to obtain a more representative picture of PA levels.

## INTRODUCTION

Chronic diseases such as cardiovascular disease and diabetes are associated with physical inactivity and are a major public health problem in developed and developing countries around the world.^1^ Increased physical activity may reduce the risk of developing such diseases.^2^ The complexity of physical activity makes it a difficult behavior to assess; therefore, the ideal assessment tool(s) should measure the activities in which specific subgroups expend the most energy.^3^ Globally, no standardized method exists for assessing physical activity; thus, it is difficult to compare physical activity levels across countries.^4^

Physical activity questionnaires are the most often used method of assessing physical activity in free-living individuals.^5^^,^^6^ Unfortunately, many such questionnaires only assess leisure physical activity, as they assume that the level of occupational physical activity is homogeneous. This assumption may be problematic when homogeneity of occupational physical activity cannot be assumed across populations. In addition, questionnaires tend to poorly capture low-intensity and unstructured physical activities, which tend to be less reproducible than moderate-to-high intensity activities such as leisure/sport or occupational activities.^5^ There are some subgroups in which a substantial amount of physical activity is low-intensity and/or unstructured and therefore unlikely to be captured by the questionnaire.^3^ A direct measure may be needed in these cases, to assess everyday and incidental activities that are difficult to recall and inaccurately captured by a questionnaire.

Concurrent use of a direct measure with a self-report measure, such as a pedometer, may yield a more representative picture of activity levels since questionnaires cannot capture low-intensity and unstructured activities.^3^ A pedometer can continuously track activity such as step counts and, unlike a questionnaire, does not rely on participant recall. Nonetheless, the pedometer cannot capture activities that are nonambulatory, such as swimming or biking, and might thus miss a substantial amount of activity. However, the use of both assessment tools (pedometer and questionnaire) may provide a more accurate measure of physical activity than a single assessment tool.

The opportunity to use both a self-report and direct assessment tool to assess physical activity in 4 racial/ethnic groups (white, African-American, Japanese-American, and Korean men) from 3 different geographic locations (Pennsylvania and Hawaii in the United States, and South Korea) was presented as part of the Electron-Beam Tomography and Risk Assessment among Japanese and US Men in the Post World War II Birth Cohort (ERA JUMP) study.^7^^–^^9^^,^^11^ Therefore, the purpose of this research was to determine the extent to which both types of assessment tools yielded the same ranking of physical activity levels (self-report vs direct measure) within the 4 racial/ethnic groups. Our hypothesis was that there would be a moderate association between the 2 types of assessment tools. Moreover, the physical activity questionnaire used in this study assesses both leisure and occupational physical activity, which allowed us to examine the contribution of occupational physical activity to overall physical activity levels.

## METHODS

### Study population

The purpose of the ERA JUMP study was to examine whether levels of subclinical atherosclerosis differ among the study populations. The participants were men 40 to 49 years of age living in Allegheny County, Pennsylvania; Honolulu, Hawaii; Kusatsu, Shiga, Japan; or Ansan City, Gyeonggi-do, South Korea. Exclusion criteria were a diagnosis of cardiovascular disease or other severe conditions, as described previously.^7^

During the period from 2002 through 2006, 1337 men aged 40 to 49 years were randomly selected to participate in the study. White (*n* = 310) and African-American (*n* = 107) men were randomly selected from voter registration lists in Allegheny County, Pennsylvania, United States.^8^^,^^9^ Japanese-American men (*n* = 303) were randomly selected from the children of members of the Honolulu Heart Program, in Honolulu, Hawaii, United States. These children were the third and fourth generation of Japanese Americans without ethnic admixture.^8^ Japanese men (*n* = 313) were randomly selected from the basic resident register of Kusatsu, Shiga, Japan,^8^ and 304 Koreans were randomly selected from the Korean Health and Genome Study, an ongoing population-based prospective cohort study, in Ansan City, Gyeonggi-Do, South Korea.^10^^,^^11^

Informed consent was obtained from all participants, and the study was approved by the Institutional Review Boards of each participating institution (University of Pittsburgh, Pittsburgh, PA, USA #0306029; Pacific Health Research Institute, Honolulu, HI, USA #03-08; Shiga University of Medical Science, Kusatsu, Shiga, Japan #12-53; and Korea University, Ansan City, Geyeonggi-Do, South Korea #ED0625) before participation in the study.

### Assessment of physical activity

Physical activity levels were assessed using both self-report and direct measurement tools. The Modifiable Activity Questionnaire (MAQ) is an interviewer-administered questionnaire that assesses both leisure and occupational activities. Before administering the MAQ, it was piloted at all the participating study sites to ensure that a comprehensive list of culturally relevant activities (leisure and occupational) was used.^3^ In addition, all study staff administering the MAQ underwent standardized training and were certified in the administration and completion of the MAQ. For this study, a past–6-month version of the MAQ was administered to all participants at their baseline clinic visit (the past–6-month MAQ is a shorter version of the original 12-month MAQ and inquires about both leisure and occupational activity performed during the 6 months before the clinic visit). Physical activity levels were calculated as the product of the duration and frequency of each activity (in hours per week), weighted by the estimate of the metabolic equivalent (MET) of that activity, and summed for all activities performed. Data were expressed as metabolic equivalent hours per week (MET hrs/wk) averaged over the past 6 months. The MAQ has been shown to be a reliable and valid assessment tool.^12^^,^^13^

Direct assessments of physical activity were obtained using the Yamax Digiwalker SW-200 (Yamasa Tokei Keiki Corporation, Tokyo, Japan) pedometer, which has been shown to be valid and reliable for assessing step counts in a variety of laboratory and field settings.^14^^,^^15^ However, it cannot assess nonambulatory activities. Participants received a pedometer, instructions for wearing the pedometer, and an activity diary at their baseline clinical examination. They were instructed to wear the pedometer during all waking hours for a period of 7 consecutive days and were permitted to remove the pedometer only for sleeping, showering, or other water activities. Participants were asked to record the number of steps taken daily, in an activity diary. At the end of the 7-day period, participants were asked to return their pedometer diary to the clinic in a postage-paid envelope. The mean number of steps the participant took per day was calculated by averaging the number of steps recorded each day during the 7-day period. Because previous research has suggested that a minimum of 3 days of activity can provide a sufficient estimate of weekly physical activity,^16^ participants with 3 or more days of data were included in the study. Steps per day averaged over the week was calculated by taking the sum of steps per day divided by the number of available days.

### Other measures

Body weight and height were measured while the participant was wearing light clothing and no shoes. Waist circumference was measured at the level of the umbilicus while the participant was standing erect. Using an automated sphygmomanometer (BP-8800; Colin Medical Technology, Komaki, Japan) with an appropriately sized cuff, blood pressure was measured in the right arm of seated participants after they had urinated and sat quietly for 5 minutes. The average of 2 measurements was recorded.

### Statistical analyses

Participants with complete physical activity data, ie, both the leisure and occupational sections of the MAQ and at least 3 days of pedometer data, were included in the analyses. Descriptive statistics were calculated for the total cohort and by race/ethnicity. All continuous data were assessed for normality. Analysis of variance (ANOVA) was used to describe and compare means among racial/ethnic groups. MAQ and pedometer data were found to be skewed and were transformed using the square root before any analyses. In post-hoc analyses, the Scheffe test was used to determine if there were any statistically significant differences in physical activity, age, or anthropometric data among the racial/ethnic groups. χ^2^ analyses were used to compare categorical variables, such as education, to identify differences among racial/ethnic groups with regard to education level. Spearman rank-order correlations were used to examine the relationship between pedometer-determined physical activity and physical activity reported on the MAQ. Additionally, tests for homogeneity of correlation coefficients were used to identify statistical differences between Spearman correlation coefficients. All statistical analyses were performed using Statistical Analysis Software, version 9.2 (SAS Institute Incorporated, Cary, North Carolina, USA). Statistical significance was defined as a 2-sided *P*-value of less than 0.05. All results are presented as mean ± SD unless otherwise noted.

## RESULTS

### Baseline characteristics of participants

A total of 855 ERA JUMP participants had complete questionnaire (MAQ) and pedometer data. Included in the final sample were 318 of 417 men (76%) in Pittsburgh (231 of whom were white and 87 were African-American), 286 of 303 (94%) Japanese-American men from Hawaii, and 251 of 304 (82%) Korean men. Japanese men from Japan were excluded from the analyses, due to the small sample size (*n* = 34) and lack of complete physical activity data. The demographic characteristics of the final sample are presented in Table 1.

**Table 1.** Descriptive characteristics of participants in the ERA JUMP study baseline cohort (*n* = 855), 2002–2006

	Pittsburgh		Hawaii	South Korea	
			
	White *n* = 231 (A)^c^	African- American *n* = 87 (B)^c^	Japanese- American *n* = 286 (C)^c^	Korean *n* = 251 (D)^c^	*P*-value^a^
Age (years)^b^	44.8 ± 2.8	44.7 ± 2.8	46.1 ± 2.8^d^	44.9 ± 2.8	<0.0001
Body mass index (kg/m^2^)^b^	28.1 ± 4.7	31.1 ± 6.6^e^	27.9 ± 4.5	24.8 ± 2.5^f^	<0.0001
Waist circumference (cm)^b^	99.4 ± 12.9	102 ± 15.2^g^	93.8 ± 11.3^h^	83.6 ± 6.7^i^	<0.0001
Hypertension (%)^j^	14.3	34.5	33.8	15.9	<0.0001
Education (%)					
<12 years	9.09	33.3	6.29	51.0	<0.0001
12–16 years	39.0	50.1	73.4	43.0	<0.0001
>16 years	52.0	16.1	20.3	5.98	<0.0001
Current smoking (%)^k^	8.23	24.1	12.2	35.1	<0.0001
Current alcohol use (%)^l^	42.9	37.9	37.1	43.0	0.4171
Physical activity^b^					
Leisure (MET hrs/wk)	9.4 ± 3.1	10.7 ± 2.8	8.9 ± 2.9	12.7 ± 2.5^m^	0.0004
Occupational (MET hrs/wk)	4.1 ± 14.2	3.4 ± 12.3	12.3 ± 15.7^n^	20.5 ± 23.6^o^	<0.0001
Total (MET hrs/wk)	20.9 ± 9.9	20.0 ± 9.1	29.4 ± 10.3	41.6 ± 17.8^p^	<0.0001
Pedometer (average steps/day)	8262.8 ± 368.6	8930.3 ± 285.6	8335.7 ± 368.6	9584.4 ± 449.4^q^	0.0001

ERA JUMP, Electron-Beam Tomography and Risk Assessment among Japanese and US Men in the Post World War II Birth Cohort; MAQ, Modifiable Activity Questionnaire; MET: metabolic equivalent.

All data are presented as mean ± SD unless otherwise noted.

^a^Comparisons among the 4 racial/ethnic groups.

^b^Physical activity data transformed from square-root transformation to original scale.

^c^Pairwise comparisons (*P* < 0.05) for age, body mass index, and waist circumference are marked as: (A vs B), (A vs C), (A vs D), (B vs C), (B vs D), and (C vs D).

^d^Japanese-American men were significantly older than white men, African-American men, Korean men.

^e^African-American men had a significantly higher body mass index than did white men and Japanese-American men.

^f^Korean men had a significantly lower body mass index than did white men, African-American men, Japanese-American men.

^g^African-American men had a significantly higher waist circumference than did Japanese-American men and Korean men.

^h^Japanese-American men had a significantly lower waist circumference than did white men.

^i^Korean men had a significantly lower waist circumference than did white men and Japanese-American men.

^j^Hypertension was defined as systolic blood pressure ≥140 mm Hg, diastolic blood pressure ≥90 mm Hg, or hypertensive medication.

^k^Current smoking was defined as smoking cigarettes during the previous month.

^l^Current alcohol use was defined as drinking alcohol on at least 2 days/week.

^m^Korean men had a significantly higher leisure activity level than did white men and Japanese-American men.

^n^Japanese-American men had a significantly higher occupational activity level than did white men and African American men.

^o^Korean men had a significantly higher occupational activity level than did white men, African-American men, and Japanese-American men.

^p^Korean men had a significantly higher total activity level than did white men, African-American men, and Japanese-American men.

^q^Korean men had significantly higher average pedometer steps/day than did white men and Japanese-American men.

Japanese-American men (age 46.1 ± 2.8 years) were slightly, but significantly, older than the white, African-American, and Korean men (*P* < 0.0001). Body mass index (BMI; 24.8 ± 2.53 kg/m^2^) and waist circumference (83.6 ± 6.74 cm) were lowest among Korean men (*P* < 0.0001). Significantly higher proportions of African-American men and Japanese-American men reported hypertension (34.5% and 33.8%, *P* < 0.0001 respectively) as compared with the white and Korean men. White men had the greatest percentage of participants with an education level greater than 16 years (52.0%, *P* < 0.0001). Korean men had the highest proportion of current smokers (35.1%, *P* < 0.0001).

### Physical activity

Mean baseline physical activity levels (MET hrs/wk) as determined by questionnaire (MAQ) and pedometer are shown in Table 1. Overall, Korean men had the highest mean MET hrs/wk of total physical activity (41.6 ± 17.8), leisure physical activity (12.7 ± 2.5), and occupational physical activity (20.5 ± 23.6) as compared with the other 3 racial/ethnic groups (*P* < 0.0001). The higher level of total physical activity in Korean men, as shown by the questionnaire, was due to their greater occupational activity. Korean men had significantly higher levels of occupational activity (20.5 ± 23.6 MET hrs/wk) than did white (4.1 ± 14.2 MET hrs/wk), African-American (3.4 ± 12.3 MET hrs/wk), and Japanese-American men (12.3 ± 15.7 MET hrs/wk). In addition, Japanese-American men reported significantly higher occupational activity as compared with white and African-American men. However, no differences were noted between total and leisure physical activity levels among these groups.

With regard to pedometer steps, similar to findings from the self-reported MAQ data, pedometer step counts were highest among Korean men (9584.4 ± 449.4 steps/day); however, post-hoc comparisons showed no statistically significant difference in step count between Korean men and African-American men. Korean men had significantly higher step counts as compared with Japanese-American and white men (*P* < 0.05).

### Physical activity correlations

We calculated Spearman rank-order correlations between the questionnaire (MAQ) and pedometer data. Table 2 presents the correlation coefficients between pedometer steps and MET hrs/wk of total physical activity, leisure physical activity, and occupational physical activity for the entire cohort and each racial/ethnic group. All correlations for total physical activity and leisure physical activity were statistically significant and positive. Correlations between occupational activity and pedometers steps were statistically significant and positive, with the exception of that for African-American men. Total physical activity (leisure and occupational activity combined) on the MAQ was more strongly associated with total pedometer steps (rho = 0.31) than were leisure activity (rho = 0.25) and occupational activity (rho = 0.26). However, tests for homogeneity of correlation coefficients showed that the values did not significantly differ.

**Table 2.** Spearman rank-order correlations between the MAQ and pedometer in the total cohort and by racial/ethnic group (ERA JUMP study, 2002–2006)

			Total pedometer steps			
		
		Total population (*n* = 855)	White (*n* = 231)	African- American (*n* = 87)	Japanese- American (*n* = 286)	Korean (*n* = 251)
MAQ	Total physical activity	0.31^a^	0.42^a,d^	0.35^b^	0.29^a,d^	0.34^a^

	Leisure physical activity	0.25^a^	0.24^b,d^	0.35^b^	0.13^c,d^	0.21^b^

	Occupational physical activity	0.26^a^	0.20^b,d^	0.10	0.26^a^	0.25^a^

ERA JUMP, Electron-Beam Tomography and Risk Assessment among Japanese and US Men in the Post World War II Birth Cohort; MAQ, Modifiable Activity Questionnaire.

^a^*P* < 0.0001; ^b^*P* < 0.01; ^c^*P* < 0.05 for Spearman rank-order correlations between MAQ domains and pedometer steps.

^d^Test for homogeneity between correlation coefficients, ie, comparison of correlation between total physical activity and pedometer steps and specific domains (leisure or occupational) of physical activity and pedometer steps, *P* < 0.05. For white men, the correlation coefficient for total physical activity and pedometer steps was significantly higher than those for the specific domains of physical activity and pedometer steps. For Japanese-American men, the correlation coefficient between total physical activity and pedometer steps was significantly higher than that between leisure physical activity and pedometer steps.

When examined separately by racial group, among white men total physical activity was more strongly correlated with total pedometer steps (rho = 0.42) than were leisure physical activity (rho = 0.24) and occupational activity (rho = 0.20). Likewise, among Japanese-American men, the correlation between total pedometer steps and total physical activity (rho = 0.29) was statistically stronger than the correlation between total pedometer steps and leisure physical activity (rho = 0.13); however, tests of homogeneity showed no significant difference between the correlations for total physical activity and occupational activity. In addition, tests of homogeneity showed no significant differences between correlations among African-American men or Korean men.

## DISCUSSION

In this culturally and geographically diverse cohort, Korean men had significantly greater physical activity levels than the other 3 racial/ethnic groups. Regardless of the assessment tool used, these findings are likely due to the fact that Korean men had the highest mean levels of leisure and occupational physical activity. There may be cultural discrepancies between the United States and South Korea in routine everyday activities such as leisure activities, transportation, domestic duties, and occupational involvement.^17^ Studying cultural differences in these various types of physical activity might reveal why Korean men were significantly more active, especially occupationally. Because we collected both leisure and occupational physical activity, we were able to see that the Korean men were occupationally more active; thus we were able to determine how this subpopulation expended most of its energy.

We noted significant positive correlations between the questionnaire and pedometer for each racial/ethnic group. In general, it appeared that total physical activity was most strongly associated with total pedometer steps, as compared with the associations of total pedometer steps with individual domains of physical activity (ie, leisure and occupational activity). However, on further inspection, we found no statistical differences between correlation values. Still, when we examined the data separately by racial and ethnic group, we did note significant differences between correlation coefficients among white men and Japanese-American men. More specifically, among white men and Japanese-American men the correlation coefficient between total pedometer steps and total physical activity on the MAQ (rho = 0.42 and 0.29, respectively) was higher than that between leisure activity and pedometer steps. Likewise, for white men, significant differences were noted for the correlations between total physical activity and pedometer steps and between occupational physical activity and pedometer steps. The stronger association between total physical activity and total pedometer steps seems logical, since pedometers measure physical activity across the entire spectrum of physical activity and are more representative of total movement. Likewise, because the pedometer does not discriminate, it measures all movement, namely, both leisure and occupational activity. If only leisure physical activity were measured, then activity performed elsewhere would be missed, resulting in a weaker correlation, as noted in this study. Assessment of the combination of leisure and occupational physical activity is more representative of total physical activity. Therefore, our findings reinforce the importance of assessing both leisure and occupational physical activity.

Previous research has suggested that minority populations in the United States tend to have lower activity levels and expend most of their energy performing low-intensity and/or unstructured activities, as compared with their white counterparts.^18^ However, our results do not appear to support this notion. African-American men did not have significantly lower levels of physical activity than whites, regardless of the assessment tool used.

This study provided a unique opportunity to examine physical activity levels by both self-report and direct methods. Both of these assessment tools have strengths and limitations, but when used in combination they may provide a more representative picture of physical activity levels because each tool has the capability of capturing different patterns and modes of activity. Moreover, the design and methods of this study allowed us to use standardized methods (pedometer and MAQ) to examine and compare physical activity levels internationally. To our knowledge, this is the first study to compare physical activity levels between US and South Korean population-based samples. Furthermore, studies using data on national physical activity among Asian-Americans combine Asian ethnic groups, because sample sizes are often insufficient for groups to be reported separately, and therefore assume that all groups are homogeneous in cultural background and immigration experience.^19^ Our investigation permitted us to focus on 1 specific Asian-American subgroup, thus allowing us to make exclusive physical activity comparisons with other US subgroups.

There are limitations that should be considered when interpreting the findings of this study. First, this study used a cross-sectional design, which limits the conclusions that can be made. Physical activity was not captured over a 12-month period, and subjects at each site had clinic visits at various time points throughout the year. While the questionnaire assessed physical activity over a 6-month period, the pedometer only assessed activity for 7 days. Because physical activity can vary due to seasonality, acute illness, and time commitments,^5^ a 12-month physical activity questionnaire, and subsequent measures with the pedometer, might provide a more representative picture of the typical physical activity behaviors of the subgroups in our investigation. Second, many pedometers, such as the one used in this study, lack an internal clock and data storage capability; thus, we had to rely on participants to record their step counts from the pedometer in their 7-day activity diary. This process may have resulted in reporting errors or missing data and consequent under/over-reporting or missing step counts.

Overall, among the 4 racial/ethnic groups, Korean men were most active, regardless of the assessment tool used, and the information provided by the questionnaire helped explain why this was so. On the questionnaire, Korean men reported significantly greater occupational physical activity than did the white, African-American, and Japanese-American men. The 2 assessment methods correlated in the relative rankings of physical activity levels between self-report and direct measures of physical activity; however, the correlations were not strong and showed that the tools were complementary rather than interchangeable.

Questionnaires such as the MAQ provide context (pattern and volume) for an activity, specifically for moderate-to-vigorous activity. However, the MAQ does not accurately assess low-intensity and unstructured activity. Therefore, supplemental use of a direct measure such as a pedometer may be required to capture activities of lower intensity, for a more accurate assessment of physical activity participation. This is particularly important when low-intensity and/or unstructured activities are the bulk of energy expenditure. Although pedometers cannot reveal the context for an activity, they can capture volume of activity in terms of step counts during ambulatory movement—which can be intermittent or low—that is not accurately recalled when assessed by questionnaire. Thus, although the use of only 1 tool does not provide a complete depiction of activity, the use of both provides a much better picture.

In conclusion, many previous studies assessed only physical activity during leisure. In the present study, the use of a questionnaire was critical in characterizing occupational activity in the Korean population and helped us understand why this group was the most active among the 4 racial/ethnic groups. If we had only assessed leisure physical activity or only used a pedometer, Korean men would still have been considered more active; however, our findings suggest that in some subpopulations it may be necessary to assess domains of physical activity beyond leisure activity. While not strongly correlated, the use of 2 assessment tools was complementary. Thus, the use of more than 1 assessment tool may provide researchers with a more representative picture of physical activity levels.
